# Teaching professionalism in medical residency programs: a scoping review protocol

**DOI:** 10.1186/s13643-020-01529-w

**Published:** 2020-12-05

**Authors:** Saeideh Ghaffarifar, Azam Asghari-Khatooni, Amirhossein Akbarzadeh, Ahmad Pourabbas, Mehran Seif Farshad, Rasoul Masoomi, Fariborz Akbarzadeh

**Affiliations:** 1 Medical Education Research Center, Health Management and Safety Promotion Research Institute, Tabriz University of Medical Sciences, Tabriz, Iran; 2 Medical Education Department, Tabriz University of Medical Sciences, Tabriz, Iran; 3 Research Center for Evidence-Based Medicine, Health Management and Safety Promotion Research Institute, Tabriz University of Medical Sciences, Tabriz, Iran; 4 Department of Medical Education, Tehran University of Medical Sciences, Tehran, Iran

**Keywords:** Professionalism, Teaching methods, Medical Residency program, Scoping review

## Abstract

**Background:**

Professionalism is a core competency of medical residents in residency programs. Unprofessional behavior has a negative influence on patient safety, quality of care, and interpersonal relationships. The objective of this scoping review is to map the range of teaching methods of professionalism in medical residency programs (in all specialties and in any setting, whether in secondary, primary, or community care settings). For doing so, all articles which are written in English in any country, regardless of their research design and regardless of the residents’ gender, year of study, and ethnic group will be reviewed.

**Methods:**

This proposed scoping review will be directed in agreement with the methodology of the Joanna Briggs Institute for scoping reviews. The six steps of Arksey and O’Malley methodological framework for conducting scoping reviews, updated by Levac et al. (Implement. Sci. 5(1): 69, 2010) will be followed. The findings from this study will be merged with those of the previous Best Evidence Medical Education (BEME) systematic review. All published and unpublished studies from 1980 until the end of 2019 will be reviewed, and the previous BEME review will be updated by the findings of the articles from the beginning of 2010 until the end of 2019. All research designs and all credible evidence will be included in this review.

**Conclusions:**

Conducting this scoping review will map the teaching methods of professionalism and will provide an inclusive evidence base to help the medical teachers in the choosing for proper teaching methods for use in their teaching practice.

**Systematic review registration:**

Not registered.

## Background

Professionalism is “the habitual and judicious use of communication, knowledge, technical skills, clinical reasoning, emotions, values, and reflection in daily practice for the benefit of the individual and community being served” [1]. It has different components such as fiduciary obligation, responsiveness to social needs, empathy, respect for others, accountability, commitment to quality and excellence, ability to deal with ambiguity and complexity, and reflection [2]. Professionalism is a necessary part of “entrustable professional activities” (EPAs). EPAs, a relatively new concept in medical education [3], are addressed by accreditation graduate medical councils such as ACGME (Accreditation Council for Graduate Medical Education) [4]. They are addressed in competency frameworks such as CanMEDS (the Canadian competency framework introduced by The Royal College of Physicians and Surgeons of Canada [5, 6] too.

Nowadays, teaching professionalism has been integrated into the mission statement of some medical schools. Professionalism is taught in some residency programs [2, 7]. Medical residents are physicians, who have completed their undergraduate program in a medical school. They are trained medical professionals, who want to complete accredited residency training program in areas such as internal medicine, surgery, radiology, or pathology. Depending on the country of study, specialty area, and off-service rotation duration, they have to complete their postgraduate specialty training in secondary, primary, or community care settings, in 3 to 6 years. After successful completion of their residency program and before working as a qualified physician, they have to pass specialty board certification exams [8].

Medical residents are among the first to visit patients. Their interpersonal relationships, quality of care provided by them, and their patients’ safety are influenced by the training on professionalism [9]. Therefore, applying valid and up-to-date methods to teach professionalism for medical residents is a vital need in today’s academic world.

Teaching methods are the ways to deliver the content of education and facilitate the learning of students [10]. Teaching methods can be student-centered or teacher-centered. They are divided into three categories: first, expository methods. They are unidirectional or passive delivery of information. Examples include lecturing or reading a book. Second is exploratory methods; they prompt exploration and discovery by learners. Exploration occurs when learners discuss or question and answer. Third is simulations, which allow practice in safe and real life-resembled situations. Role playing is a good example of simulation [11]. Teaching methods of professionalism include role modeling, feedback, group discussion, case-based discussion, reflection, holding ethical rounds, and reports [12]. They are not limited to these methods. New methods, such as teaching through social media [13] and learning management systems [14], have been employed to teach professionalism over the last decade.

The importance of teaching professionalism is highlighted in a narrative review in 2014 [7]. All teaching methods of professionalism, which have been reported in published articles between 1999 and 2009, are reviewed in a Best Evidence Medical Education (BEME) systematic review [12].

While being important, teaching professionalism is challenging too. Many medical teachers are not trained enough to teach professionalism [15]. They personally choose to employ various methods to teach professionalism. This is why they have to keep themselves updated on the methods of teaching professionalism.

Some studies (no systematic review) on teaching professionalism across surgery, orthopedic, and ophthalmology residency programs have been already conducted [16–19].

In a systematic review by Berger and colleagues, professionalism curricula in postgraduate medical education (PGMED) are reviewed in 50 included interventional studies. The authors have searched only three databases, and reviewing the teaching methods of professionalism has been a small part of this review. Method of teaching professionalism has not been the subject of research in 17 out of 50 included studies in this review and some new methods of teaching professionalism such as teaching through social media or learning management systems are not included in the review. Except for teaching methods of professionalism, curricula duration, effectiveness, and assessment modalities are evaluated in this review too. As the authors of this review have concluded, finding best practices based on synthesizing previous findings, which were very heterogeneous, was difficult. It is also concluded that “even simple, short teaching sessions” would affect professionalism [6].

Therefore, in order to help the medical teachers to keep themselves updated on methods of teaching professionalism, it was intended to conduct a scoping review focused on methods of teaching professionalism in medical residency programs, in all databases, regardless of the research design of the included studies. For this purpose, all published and unpublished studies from 1980 until the end of 2019 will be reviewed, and the previous BEME review will be updated by the findings of the articles from the beginning of 2010 until the end of 2019. The findings from this study will be merged with those of the previous BEME systematic review. As different medical specialties are of different nature, while reviewing teaching methods of professionalism, the methods will be systematically categorized by different specialty areas of residency training.

Conducting this scoping review will map the teaching methods of professionalism, divided by specialty areas in PGME and will provide an inclusive evidence base to help the medical teachers in the choosing for proper teaching methods for use in their teaching practice and make even a little change on promoting professionalism in their own specialty area.

## Methods

This proposed scoping review will identify and map the available and emerging evidence on the topic of teaching professionalism in medical residency programs. It will be directed in agreement with the methodology of the Joanna Briggs Institute for scoping reviews [20]. The six steps of Arksey and O’Malley methodological framework for conducting scoping reviews updated by Levac et al. will be followed [21].

### Step 1: identifying the research question(s)

This review will be guided by the following research questions:

1. “What methods have been used to teach professionalism in each specialty area of medical residency programs?”
2. What methods have been used to teach professionalism for medical residents in each culture?
3. What methods have been used to teach professionalism for medical residents in each setting?
4. How many medical residents have been trained about professionalism in one session, by each teaching method?
5. What resources have been used to teach professionalism to medical residents, by each teaching method?
6. What role(s) have teachers played in applying each method to teach professionalism to medical residents?
7. What skills were applied to employ each method to teach professionalism for medical residents?

### Step 2: identifying relevant studies

For this review, the following general keywords are identified: professionalism, medical ethics, professional role, teaching, education, hospitals, health care system, health system, primary care setting, secondary care setting, community care setting, teaching method, educational method, residency program, medical training, medical residents, and resident doctors. Specific keywords, which will be used during literature search, will be reported later in the final review.

A broad search on teaching professionalism in postgraduate medical education will be conducted. All the articles and studies will be filtered, and only those related to medical residency programs will be selected for the next steps.

The results will be merged with all related studies which were reviewed in BEME Guide Number 25 [12].

Both published and unpublished studies (gray literature) will be considered in the search strategy. An initial search has been conducted in three main databases (Medline, EMBASE, and ERIC) until December 2019. A sample of our search strategy is added as Additional file 1. The search strategy will be refined if it is necessary and will be updated for the final review. So, according to findings of the primary search, appropriate studies on teaching professionalism will be identified. Based on the text words in titles and abstracts and index terms, a full search strategy will be developed to look for published studies on MEDLINE (through PubMed), Scopus, EMBASE, PsycINFO, Cochrane, and Web of Science. Complementary search strategies will be described in the final report of the review. The same search strategy, keywords, and index terms will be employed in all databases. Articles published in 2018 and 2019 in renowned medical education journals, including medical teacher, medical education, academic medicine, clinical teacher, and teaching and learning in medicine, will be searched manually. Websites of different associations for medical training such as “The International Association for Medical Education (AMEE)” [22], “The Association for the Study of Medical Education (ASME)” [23], “World Federation for Medical Education (WFME)” [24], or ACGME (Accreditation Council for Graduate Medical Education) [25] will be searched as well. In addition to searching electronic databases and hand searching, reference lists of the included articles will be explored. Dissertations and theses on ProQuest, GreyNet, and Google Scholar will be searched to find the gray literature in this regard. Authors of primary studies will be contacted for clarification or missing information. Leading authors in the field of teaching professionalism will be contacted. Search strategy in Ovid MEDLINE® database is shown in Table 1. A librarian with the relevant experience and knowledge will conduct all database searches and manage records and data throughout the review. The search strategy will be peer-reviewed using the Peer Review of Electronic Search Strategies (PRESS) checklist [26].

**Table 1:** Search strategy in Ovid MEDLINE(R) and Epub Ahead of Print, In-Process & Other Non-Indexed Citations, Daily and Versions (R)

1	exp Professionalism/
2	Professionalism.tw.
3	“professional behavio?r”.tw.
4	“professional practice”.tw.
5	1 or 2 or 3 or 4
6	teaching.tw.
7	education.tw.
8	training.tw.
9	instruction.tw.
10	learning.tw.
11	6 or 7 or 8 or 9 or 10
12	exp Education, Medical, Graduate/
13	Graduate Medical Education.tw.
14	medical.tw.
15	residen$.tw.
16	12 or 13 or 14 or 15
17	5 and 11 and 16

*1946 to March 13, 2020 (search date 15 March 2020)

### Step 3: study selection

After completing the search and loading all studies into EndNote X8, duplicates will be removed. In dealing with reviews that included duplicate original studies, if the original article is evaluated correctly in the review article, the review article will be used in the study instead of the original article. Moreover, secondary analyses related to a trial will not be included in the study. Secondly, assuming that a trial study may have been included in two different review studies, the occurrence of such a hypothetical case and not being able to delete that duplicate trial will be mentioned as a limitation of the study because we cannot disregard one of those two reviews because of the duplication of that trial. Inclusion and exclusion criteria for selecting articles are shown in Table 2. Two independent reviewers will simultaneously screen titles and abstracts. Through such a simultaneous screening, a maximum number of relevant studies will be identified. Reviewers will be blind to each other’s decision. In case of any inconsistency between reviewers, the disagreement will be discussed in order to be resolved by a third reviewer. The full text of possibly pertinent studies will be recovered and assessed to probe for inclusion criteria for the review by two independent reviewers. The reviewers will provide explanations about the logic of excluding any full-text article. Again, reviewers will independently review full articles, and final decision will be determined by a third reviewer.

**Table 2:** Inclusion and exclusion criteria for selecting articles

Inclusion criteria: studies …	Exclusion criteria: studies ….
- Have been reported from the beginning of 2009 until the end of 2019 - In English language - All research designs - All methods of teaching professionalism - On residency groups in medicine - In all residency specialty programs - All departments, disciplines, and settings - In all years of residency programs - On any gender of medical residents - In all countries and ethnic groups	- In languages other than English - On nursing residency programs - On dentistry residency programs - On residency programs of medical groups other than medicine (rehabilitation, physiotherapy, etc.) - On undergraduate medical programs - On continuous medical education (CME) - On personal development plans - Which have focused on only assessment of professionalism in medical residency programs

The search results will be fully reported, and the final report will be shown in a Preferred Reporting Items for Systematic Reviews and Meta-analyses (PRISMA) flow diagram [27].

### Step 4: charting the data

Detail of data will be extracted in accordance with the JBI-recommended approach [20]. Data will be extracted by two independent extractors (blind to each other). A pilot form, with minimum requirements, is considered for data extraction. Based on the findings from the literature review, other necessary details will be added to the form. In order to standardize the form, consultation with experts in the field will be adopted. The data extraction tool for this protocol is presented in Table 3. The results will be presented in the form of a single table with two separate sections for review articles and original articles. Some specific items related to articles will be identified with specific symbols in the table.

**Table 3:**
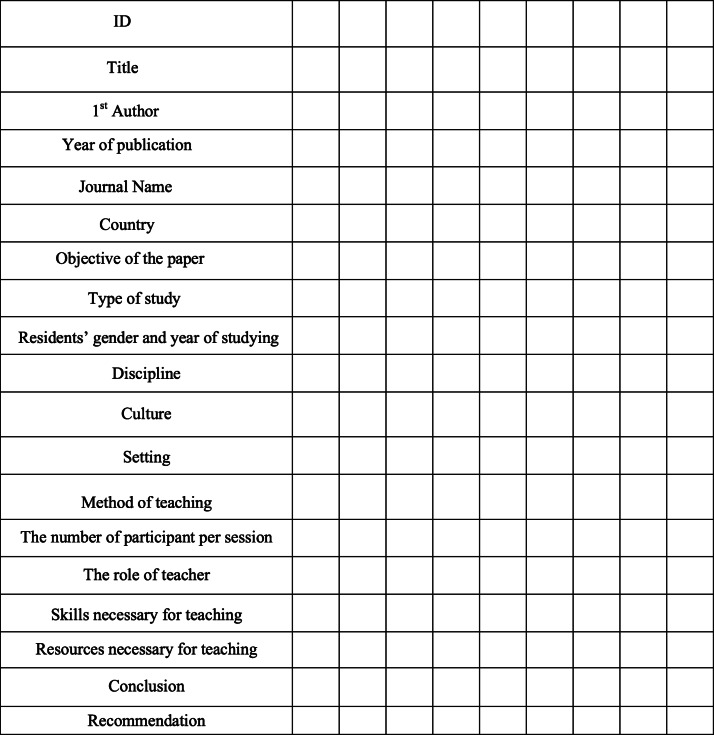
Data extraction table

In this review, except for the data including the name of the first author, journal name, publication year, country of study, residents’ characteristics (PGY) including year of residency, objective of study, type of study, conclusions, and recommendations, qualitative data will be extracted, and the findings will be grouped into seven key variables: training discipline, culture, method of teaching professionalism, setting of teaching, number of participants in each educational session, the role of the teacher, necessary skills for teaching, resources used.

All data on teaching methods of professionalism in medical residency programs will be charted and summarized in relation to the objective of the study.

### Step 5: collating, summarizing, and reporting results

A total number of searched studies and selected ones will be reported. The decision process will be described. The flow of selection process will be reported using a PRISMA flow diagram [27]. Results from primary search, deletion of duplicate studies, selection of studies, and additional search results (gray literature and reference search of selected studies, and author contacts) will be added in the decision process and a final summary will be presented.

A narrative description of the main objective in teaching methods of professionalism to medical residents will be presented.

The results will be classified under categories such as residents’ year of study and studying discipline and setting of training. Charts or tables will be used to present the results such as distribution of the teaching methods by residents’ year of study or etc. If quantitative synthesis was not possible, the results would be only described.

Implications of the study finding for both practice and research will be identified.

### Step 6: consultation

Primary findings will be used as a basis for consultation with both medical teachers and residents. By such a consultation, search strategies will be refined, and opportunities for knowledge transfer will be provided. The findings of this review will be shared at educational conventions and conferences. The report of the review as well as a peer-reviewed article will be published as well.

### Assessment of bias

Questions in this review do not relate to quality assessment debates. So, due to the nature of the aims of this review, risk of bias of the included evidence will not be evaluated as it is usual in most scoping reviews [28]. Moreover, as this review is a scoping one, reviewing the effectiveness of the teaching methods and their values will not be the primary aim of this review. Hence, no publication bias across studies or selective reporting within studies will be investigated by the reviewers; however, if in studies such as trials, the effect of a teaching method has been assessed and reported, the effects of different teaching methods will be categorized and reported. For doing so, quality assessment of quantitative and qualitative studies will be done by EPHPP checklist [29]. The quality studies will be grouped with each other, and necessary analysis will be done later, if applicable. Such a step would be considered as the inductive part of data extraction. Such an analysis will increase the use of finding by medical teachers in their daily practices.

## Discussion

In this scoping review, the available evidence and innovative practices on the topic of the methods to teach professionalism in medical residency programs will be identified and mapped. By synthesizing the evidence, research gaps in the existing literature will be recognized and the emerging research priorities will be consulted with stakeholders. Limitations of conducting this review, practical implications, and recommendations for future research will be reported in the discussion of the protocol’s article.

## Supplementary Information

**Additional file 1.** A sample of search in Ovid MEDLINE(R) and Epub Ahead of Print, In-Process & Other Non-Indexed Citations, Daily and Versions(R)

## Data Availability

The data sets generated and/or analyzed during the current study will not be publicly available before publication of its pertinent manuscripts, but are available from the corresponding author on reasonable request.

## Abbreviations

EPAs: Entrustable professional activities
ACGME: Accreditation Council for Graduate Medical Education
BEME: Best Evidence Medical Education
AMEE: The International Association for Medical Education
ASME: The Association for the Study of Medical Education
WFME: World Federation for Medical Education
