# Supplementary material for: Teaching professionalism in medical residency programs: a scoping review protocol
Source: Syst Rev. 2020 Dec 5;9:281. doi: 10.1186/s13643-020-01529-w (PMC7719236; doi:10.1186/s13643-020-01529-w)
Supplement: Supplementary file 1 — Additional file 1. A sample of search in Ovid MEDLINE(R) and Epub Ahead of Print, In-Process & Other Non-Indexed Citations, Daily and Versions(R) [file 13643_2020_1529_MOESM1_ESM.docx]

**A sample of search in Ovid MEDLINE(R) and Epub Ahead of Print, In-Process & Other Non-Indexed Citations, Daily and Versions(R)**

| 1 | exp Professionalism/ | 1030 |
| --- | --- | --- |
| 2 | Professionalism.tw. | 6992 |
| 3 | "professional behavio?r".tw. | 875 |
| 4 | "professional practice".tw. | 4794 |
| 5 | 1 or 2 or 3 or 4 | 12716 |
| 6 | teaching.tw. | 136089 |
| 7 | education.tw. | 431919 |
| 8 | training.tw. | 393866 |
| 9 | instruction.tw. | 26378 |
| 10 | learning.tw. | 282034 |
| 11 | 6 or 7 or 8 or 9 or 10 | 1076681 |
| 12 | exp Education, Medical, Graduate/ | 68282 |
| 13 | Graduate Medical Education.tw. | 5402 |
| 14 | medical.tw. | 1125256 |
| 15 | residen$.tw. | 235644 |
| 16 | 12 or 13 or 14 or 15 | 1350723 |
| 17 | 5 and 11 and 16 | **2765** |

* 1946 to March 13, 2020(Search Date: 15 March 2020)
